# Phospho-ΔNp63α/Rpn13-dependent regulation of LKB1 degradation modulates autophagy in cancer cells

**DOI:** 10.18632/aging.100249

**Published:** 2010-12-20

**Authors:** Yiping Huang, Edward A. Ratovitski

**Affiliations:** Department of Dermatology, Johns Hopkins University School of Medicine, Baltimore, MD 21231, USA

**Keywords:** p63, LKB1, oncogene, tumor suppressor, protein degradation, autophagy, chemoresistance

## Abstract

Oxidativue stress was shown to promote the translocation of Ataxia-telangiectasia mutated (ATM) to cytoplasm and trigger the LKB1-AMPK-tuberin pathway leading to a down-regulation of mTOR and subsequently inducing the programmed cell death II (autophagy). Cisplatin was previously found to induce the ATM-dependent phosphorylation of ΔNp63α in squamous cell carcinoma (SCC) cells. In this study, phosphorylated (p)-ΔNp63α was shown to bind the ATM promoter, to increase the ATM promoter activity and to enhance the ATM cytoplasmic accumulation. P-ΔNp63α protein was further shown to interact with the Rpn13 protein leading to a proteasome-dependent degradation of p-ΔNp63α and thereby protecting LKB1 from the degradation. In SCC cells (with an altered ability to support the ATM-dependent ΔNp63α phosphorylation), the non-phosphorylated ΔNp63α protein failed to form protein complexes with the Rpn13 protein and thereby allowing the latter to bind and target LKB1 into a proteasome-dependent degradation pathway thereby modulating a cisplatin-induced autophagy. We thus suggest that SCC cells sensitive to cisplatin-induced cell death are likely to display a greater ratio of p-ΔNp63α/non-phosphorylated ΔNp63α than cells with the innate resistant/impaired response to a cisplatin-induced cell death. Our data also suggest that the choice made by Rpn13 between p-ΔNp63α or LKB1 to be targeted for degradation is critical for cell death decision made by cancer cells in response to chemotherapy.

## INTRODUCTION

Cisplatin is the most applicable drug for treating various human cancers, however, its efficiency is limited due to development of drug resistance by tumor cells [1-3]. Cisplatin-induced programmed cell death is associated with expression of specific “cell death” genes and down regulation of “survival” genes [1-3]. Failure of cancer cells to maintain expression of the former genes may be an important factor in cisplatin resistance [1-3]. Previous reports from our research team emphasized the intriguing link between p63 regulatory roles in gene transcription and protein stability, and resistance of tumor cells to cisplatin chemotherapy [3-5]. *P53* homolog *p63* is a novel transcription factor implicated in regulation of genes involved in DNA damage response and chemotherapeutic stress in tumor cells [3-6]. Due to the two independent promoters, *p63* gene encodes two types of protein isotypes, with the long transactivation (TA)-domain and with the short TA- domain [3, 6]. The latter is designated as ΔNp63α. Due to several alternative-splicing events p63 produces three isotypes with the various length of the carboxyl terminus (α, β and γ). ΔNp63α is the longest and is the most predominant isotype expressed in squamous cell carcinoma (SCC) cells [3-5].

ΔNp63α is phosphorylated by the Ataxia-telangiectasia mutated (ATM)-dependent mechanism following cisplatin treatment, functioning as a pro-survival factor in SCC cells [4,5]. From the other hand, the ΔNp63α ability to activate ATM transcription thereby supports a feedback-regulatory mechanism [7]. However, whether this transcription factor needs to undergo phosphorylation in order to activate ATM transcription remains unclear. Moreover, ATM was shown to translocate to cytoplasm where it phosphorylates LKB1 kinase [8,9] subsequently leading to an autophagic process through an AMPK/mTOR signaling pathway [10-12]. Finally, cisplatin was shown to induce the phospho (p)-ΔNp63α-dependent regulation of the regulatory particle non-ATPase subunit (Rpn)-13 gene transcription thereby contributing to cell death pathway of tumor cells [13]. Here, we report that upon cisplatin exposure, SCC cells displayprotein complex formations between Rpn13, ΔNp63α or LKB1 leading to a proteasome-dependent degradation of p-ΔNp63α or LKB1 by binding to Rpn13 in turn leading to autophagic-related chemosensitivity or chemoresistance.

**Figure 1.**
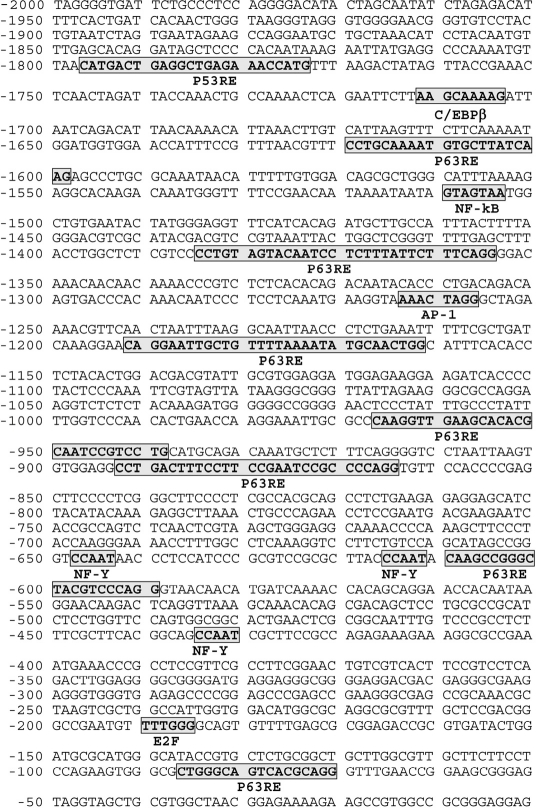
Schematic representation of ATM (2000 bp) promoter Putative cognate sequences for transription factors are bolded, bordered and shadowed. Several p63 responsive elements (RE) were found in the ATM promoter sequence.

## RESULTS

### P-ΔNp63α regulates the ATM transcription

ΔNp63α was previously found to activate the ATM transcription in human keratinocytes [7]. This transcription factor was shown to induce the ATM transcription through the CCAAT element found in the human ATM promoter (Fig. 1). As shown in Figure 1, the ATM promoter contains a few p63 responsive elements (RE) along with E2F and NF-Y cognate sequences, where latter one specifically binds to the CCAAT element playing a critical role for p-ΔNp63α dependent regulation of transcription [5]. Although, previous report supports the ability of ΔNp63α to induce ATM transcription [7], it is unclear whether the ΔNp63α phosphorylation is needed for ATM transcriptional regulation. To access the role for p-ΔNp63α in the regulation of ATM expression under DNA damage, we employed the cellular model, isogenic SCC clones, which contain the genomic copy of wild type ΔNp6α or ΔNp63α-S385G. The latter protein displays an altered ability to be phosphorylated by ATM kinase upon cellular response to cisplatin treatment [4,5]. These clones were used as tools to examine the role for phosphorylation of ΔNp63α in transcriptional regulation of gene expression and in the cellular response to chemotherapeutic treatment allowing us to define novel gene targets involved in cisplatin-mediated resistance [4,5].

**Figure 2.**
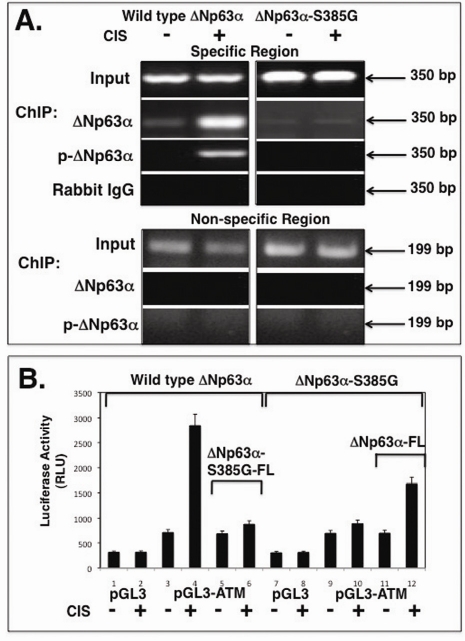
Binding of the p-ΔNp63α protein to the ATM promoter in vivo Wild type ΔNp63α cells (left panels) and ΔNp63α-S385G cells (right panels) were exposed to a control medium and 10μg/ml cisplatin for 24h. (**A**) ChIP assay of a specific region of the ATM promoter with anti-p-ΔNp63a antibody and anti-DNp63 antibody. As negative controls, we used ChIP of the ATM promoter specific region with rabbit immunoglobulins (IgG) and ChIP of the ATM promoter non-specific region with anti-p-ΔNp63α antibody as indicated. (**B**) Luciferase reporter assay. Both types of cells were transfected with 100 ng of the promoter-less pGL3 plasmid or pGL3-ATM (1259bp) promoter plasmid along with 1 ng of the pRL-SV40 plasmid for 24h. Cells were also transfected with 100 ng of the ΔNp63α-FL (Flag) or ΔNp63α-S385G-FL expression cassettes, as indicated. Cells were exposed to control medium (Con) and 10 μg/ml cisplatin (CIS) for 24h. Luciferase reporter assays were conducted in triplicate (± SD are indicated, p<0.05) as described in the Materials and methods. Firefly luciferase activity values were normalized by Renilla luciferase values.

Using ChIP analysis with antibodies to ΔNp63 and p-ΔNp63α, we found that cisplatin exposure led to an increase of the p-ΔNp63α binding to the ATM promoter in wild type ΔNp63α cells, while there is no such binding found in ΔNp63α-S385G cells (Fig. 2A). Furthermore, p-ΔNp63α binding was associated with the specific region of the ATM promoter containing NF-Y/ CCAAT element (Fig. 2A). There is no ΔNp63α binding was found in the non-specific region of the ATM promoter (Fig. 2A). The quantitative analysis of p-ΔNp63α binding by qPCR showed that the cisplatin treatment dramatically induced p-ΔNp63α binding to the ATM promoter (by ~14.5 ± 1.3 fold) in wild type ΔNp63α cells only (Suppl. Fig. S1).

We further tested whether the ΔNp63α phosphorylation affects the ability of ΔNp63α to induce the ATM promoter-driven luciferase reporter. Wild type ΔNp63α cells and ΔNp63α-S385G cells were transfected with the promoter-less pGL3-Luc and pGL3-ATM (1259)-Luc plasmids followed by the exposure of cells to a con-trol medium or 10μg/ml cisplatin for 24h. We showed that the cisplatin treatment significantly increased the ATM promoter-driven luciferase activity in wild type ΔNp63α cells (by ~4.01 ± 0.34 fold), while no such effect (by ~1.06 ± 0.12) was observed in ΔNp63α-S385G cells upon cisplatin exposure (Fig. 2B). In addition, 100 ng of the ΔNp63α-FL expression construct and ΔNp63α-S385G-FL construct was introduced into ΔNp63α-S385G cells and wild type ΔNp63α cells, respectively (Fig. 2B). We observed that ΔNp63α-S385G-FL markedly attenuated the cisplatin-mediated activation of the luciferase activity (by ~1.28 ± 0.12 fold) in wild type ΔNp63α cells, while ΔNp63α-FL increased this activity (by ~2.39 ± 0.21) in ΔNp63α-S385G cells (Fig. 2B).

### P-ΔNp63α induces ATM-mediated LKB1-mTOR pathway

**Figure 3.**
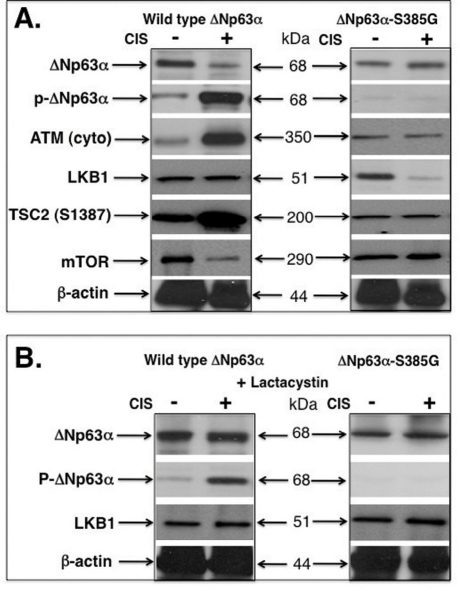
Cisplatin induces the p-ΔNp63α and LKB1 protein levels in SCC cells Wild type ΔNp63α cells and ΔNp63α-S385G cells were exposed to control media and 10μg/ml cisplatin for 24h. Protein levels were tested with indicated antibodies. Cytoplasmic (cyto) protein levels were tested with the anti-β-actin antibody. (**A**) No lactacystin treatment. (**B**) With lactacystin treatment.

Recent seminal report by Dr. Cheryl Walker' group clearly demonstrated the stress-dependent export of ATM from nucleus to cytoplasm subsequently leading to the LKB1 phosphorylation followed by tuberin (TSC2) activation and down-regulation of mTOR [8,9]. Since ΔNp63α induces ATM expression [7], we suggested the potential role for p-ΔNp63α in ATM regulation, ATM translocation to cytoplasm and ATM-dependent triggering of the LKB1-TSC2-mTOR pathway. We treated wild type ΔNp63α cells and ΔNp63α-S385G cells with a control medium and 10μg/ml cisplatin for 24h. We then found that the cisplatin exposure induced the ΔNp63α phosphorylation leading to reduction of ΔNp63α protein levels (Fig. 3A). At the same, the levels of cytoplasmic ATM and activated TSC2 were significantly increased, while mTOR protein levels were decreased in wild type wild type ΔNp63α cells upon cisplatin exposure (Fig. 3A, left panel). No such changes were observed in ΔNp63α-S385G cells (Fig. 3A, right panel). However, we showed that the LKB1 levels were decreased in ΔNp63α-S385G cells after cisplatin treatment suggesting the possibility for LKB1 to be degraded (Fig. 3A, right panel). To further examine this hypothesis, we exposed cells to lactacystin, the 26S proteasome inhibitor [13]. We thus found that the lactacystin treatment (25 mM for 12h) rescued ΔNp63α degradation in wild type ΔNp63α cells, and LKB1 degradation in ΔNp63α-S385G cells (Fig. 3B) suggesting the critical involvement of the 26S proteasome machinery.

### Rpn13 binding promotes a proteasome-dependent degradation of either p-ΔNp63α or LKB1

A regulatory particle non-ATPase subunit (Rpn)-13 was shown to function as a 19S proteasome cap-associated protein, acting as an ubiquitin receptor recruiting the deubiquitinating enzyme UCH37 to the 26S proteasome [13-18]. We previously found that the cisplatin treatment induced Rpn13 transcription by p-ΔNp63α and subsequently increased the physical interaction of Rpn13, UCH37 and NOS2 proteins leading to an essential degradation of the latter through a proteasome-dependent mechanism in SCC cells [13].

**Figure 4.**
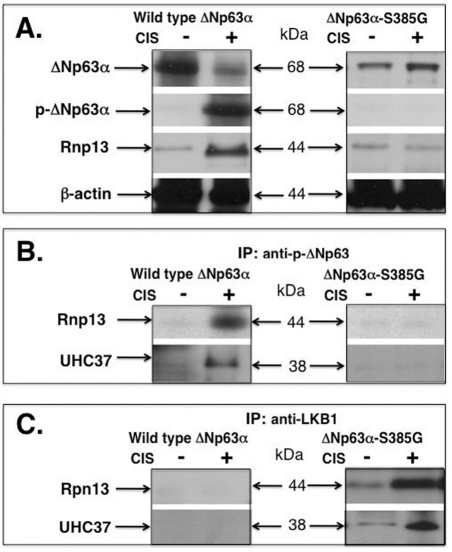
Cisplatin induces a protein complex formation between p-ΔNp63α and Rpn13 in wild type ΔNp63α cells and between Rpn13 and LKB1 in ΔNp63α-S385G cells Wild type ΔNp63α cells and ΔNp63α-S385G cells were exposed to control media and 10μg/ml cisplatin for 24h. Immunoprecipitation (IP) was performed with indicated antibodies and the protein levels were tested with indicated antibodies. (**A**) Immunoblotting. (**B**) and (**C**) Immino-precipitation.

In this study, we examined whether the Rpn13-dependent mechanism is implicated in down-regulation of the ΔNp63α protein or LKB1 protein in SCC cells upon cisplatin exposure. First, we showed that the cisplatin treatment reduced the ΔNp63α protein level, while induced the ΔNp63α phosphorylation level in wild type ΔNp63α cells (Fig. 4A, left panel). At the same time, cisplatin up-regulated Rpn13 protein level in wild type ΔNp63α cells (Fig. 4A, left panel). However, ΔNp63α and Rpn13 levels were not changed in ΔNp63α-S385G cells after cisplatin exposure, while no p-ΔNp63α was detected (Fig. 4A, right panel). We further showed that the cisplatin exposure induced a complex formation between Rpn13, UCH13 and p-ΔNp63α proteins in wild type ΔNp63α cells (Fig. 4B, left panel), while no such complexes were observed in ΔNp63α-S385G cells (Fig. 4B, right panel). We next found that the cisplatin treatment led to a physical association between Rpn13, UCH37 and LKB1 proteins in ΔNp63α-S385G cells (Fig. 4C, right panel), while no similar protein complexes were detected in wild type ΔNp63α cells (Fig. 4C, left panel).

### P-ΔNp63α enhances the autophagic process in SCC through a LKB1-dependent pathway

Accumulating evidence supports the notion that stress induces autophagic-related characteristics through the LKB1-AMPK-TSC-mTOR pathway [10-12]. We thus examined whether cisplatin treatment promotes the autophagy, and whether LKB1- or Rpn13- dependent mechanisms play any role in it. Microtubule-associated protein light chain 3 (LC3B), a mammalian homolog of yeast Atg8, has been used as a specific marker to monitor autophagy [19]. Upon induction of autophagy, the cytosolic form LC3B (LC3B-I) is conjugated to phosphatidylethanolamine (conversion into LC3B-II) and targeted to autophagic membranes.

**Figure 5.**
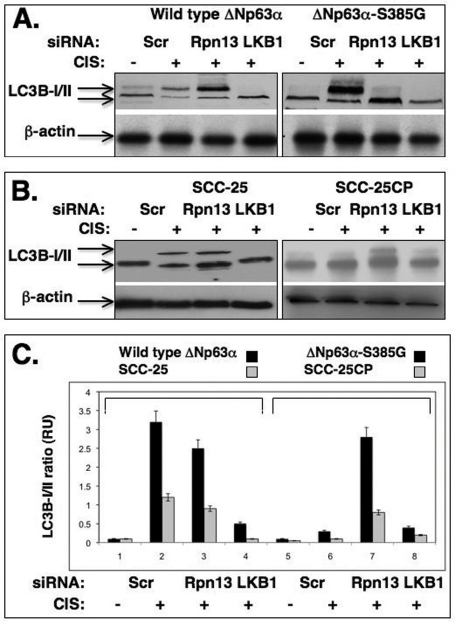
Cisplatin induces the autophagic process through a LKB1 up-regulation (**A**) Wild type ΔNp63α cells and ΔNp63α -S385G cells and (**B**) Sensitive (SCC-25) and resistant (SCC-25CP) squamous carcinoma cells were exposed to control media and 10μg/ml cisplatin for 24h. Cells were transiently transfected with scrambled siRNA, siRNA against Rpn13 or LKB1. Cells were grown up in the presence of lyzosomal protease inhibitors (10 μg/ml of both E64d and pepstatin A). Protein levels of autophagic markers were analyzed by immunoblotting with indicated antibodies. β-actin expression was used as a loading control. (**C**). Quantitative analysis of LC3B -I/II ratio. Immunoblots were scanned using PhosphorImager (Molecular Dynamics) and quantified by Image-Quant software version 3.3 (Molecular Dynamics). Values of LC3B-II were expressed as a portion of LC3B-I values defined as 1. The LC3B-II/LC3B-I ratios were plotted as bars using the Microsoft Excel software with standard deviations (± SD, p>0.05) resulting from three independent experiments and three individual measurements of each experiment. Black bars represent the set of wild type ΔNp63α/ΔNp63α-S385G cells, while grey bars represent a set of SCC-25/SCC-25CP cells.

The S385G mutation shown to impair the ability for ΔNp63α to be phosphorylated by ATM in SCC cells upon cisplatin treatment is a superficial tool providing a suitable model system to investigate a potential relationship between ΔNp63α and ATM-dependent phosphorylation. Thus, we used this model to assess a role for ATM-dependent phosphorylation of the ΔNp63α in cisplatin chemoresistance of SCC cells. Wild type ΔNp63α cells and ΔNp63α-S385G cells were transiently transfected with scrambled siRNA, siRNA against Rpn13 or LKB1 for 24h and then exposed to control media or 10μg/ml cisplatin for 24h in the presence of 10μg/ml of lyzosomal protease inhibitors, E64d and pepstatin A, as recommended elsewhere [19]. By immunoblotting with an antibody against an autophagosome marker, LC3B-I (16 kDa) and its conversion variant LC3B-II (14 kDa), we observed the cisplatin-induced autophagy-related changes of LC3B expression in SCC cells (Fig. 5). We found that wild type ΔNp63α cells (which support the ΔNp63α phosphorylation in response to cisplatin) displayed a marked expression of LC3B-II, while siRNA against LKB1 dramatically reduced this effect. Interestingly, siRNA against Rpn13 had a minimal effect on the cisplatin-induced LC3B-II activation (Fig. 5A, left panel). From the other hand, cisplatin treatment failed to induce autophagic changes in LC3B expression in ΔNp63α-S385G cells (Fig. 5A, right panel). Intriguingly, siRNA against Rpn13 markedly increased the level of the LC3B-II autophagic marker in ΔNp63α-S385G cells (Fig. 5A, right panel) suggesting the Rnp13-dependent regulation of LKB1 protein levels in these cells.

We also employed a set of SCC cells displaying sensitivity or resistance to cisplatin (SCC-25 and SCC-25CP, respectively) as reported elsewhere [20]. We first examined whether SCC-25 and SCC-25CP cells expressed ΔNp63α or p-ΔNp63α in response to cisplatin treatment. We found that after cisplatin exposure, resistant SCC-25CP cells, indeed, express far less of the p-ΔNp63α protein than their sensitive counterpart, SCC-25 (Suppl. Fig. S2). We further found that, in constrast to SCC-25 cells, cisplatin reduced the LKB1 protein levels in SCC-25CP cells (Suppl. Fig. S2) suggesting that the greater ratio between non-phosphorylated ΔNp63α and p-ΔNp63α might be an important factor contributing to LKB1 reduction and is likely to play a role in cisplatin resistance displayed by SCC-25CP cells.

**Figure 6.**
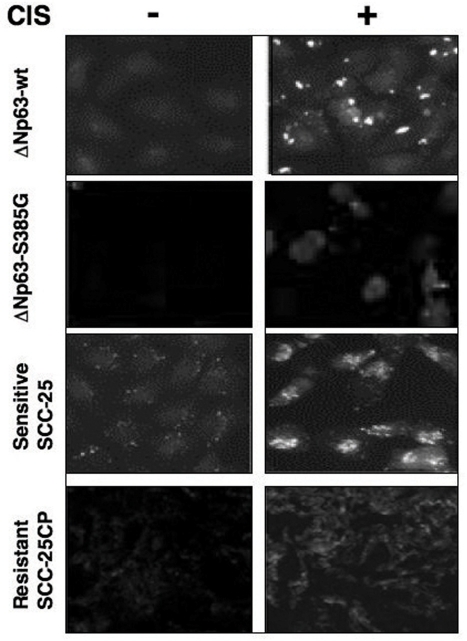
Immunofluorescence staining of LC3B expression in squamous carcinoma cells upon cisplatin exposure Sets of wild type ΔNp63α/ΔNp63α-S385G cells and SCC-25/SCC-25CP cells were exposed to control media and 10μg/ml cisplatin for 24h. Cells were stained with a polyclonal antibody against MAP LC3α/β (1:100), and then photographed under fluorescent microscope.

We further found that cisplatin induced the LC3B-II expression in sensitive SCC-25 cells, while siRNA against LKB1 significantly inhibited this expression, and siRNA against Rpn13 had only a minimal effect on the LC3B-II level reduction (Fig. 5B, left panel). In the case of resistant SCC-25CP cells, one could see no cisplatin-induced LC3B-II up-regulation, however siRNA against Rpn13 promoted a significant increase of this autophagic marker (Fig. 5B, right panel). The quantitative analysis of the LC3B-I/LC3B-II ratio (Fig. 5C) led us to the same conclusion. Using immunofluorescence analysis, we further examined the expression of LC3B-II isotype in SCC upon cisplatin exposure and observed the clustering (“punctuated appearance”) of the membrane-associated protein, MAP LC3α/β (LC3B), as previously described elsewhere [19,21]. We also observed that cisplatin-resistant cells showed much lesser autophagic-related levels of LC3B-II expression than cisplatin-sensitive cells (Fig. 6) suggesting the critical role for autophagy in tumor response to a chemotherapeutic treatment.

## DISCUSSION

An optimal cellular response to DNA damage/stress (ionizing radiation, oxidative stress, chemotherapeutic drugs, UV radiation, nutrient deprivation, and hypoxia) requires repair of damage and coordination of critical cellular processes such as transcription, translation, metabolism, and control of cell survival through an apoptosis or autophagy [22-26].

Emerging evidence supports the notion that the cisplatin-induced autophagy plays a central role in tumor cell resistance to platinum-based therapy [27-29]. A dose- and time-dependent induction of autophagy observed in tumor cells following cisplatin treatment is evidenced by up-regulation Beclin-1 and cisplatin-triggered activation of AMPK pathway leading to a subsequent suppression of mTOR activity [28]. Autophagy is also shown to delay apoptosis in renal tubular epithelial cells exposed to cisplatin cytotoxicity [30,31]. The switch from autophagy to apoptosis suggests that autophagy induction mediates a pre-apoptotic lag phase observed in renal tubular cells exposed to cisplatin supporting the idea that autophagy mounts an adaptive cell response that delays apoptosis and might contribute to a cisplatin resistance in other cellular systems including cancer [30,31].

A few oncogenes (e.g. phosphatidylinositol 3-kinase, activated AKT1) inhibit autophagy, while numerous tumor suppressors (e.g. BH3-only proteins, death-associated protein kinase-1, PTEN, tuberous sclerosic complex 1 and 2, TSC1 and TSC2 and LKB1/STK11) induce autophagy [32]. As known guardians of genome integrity, p53 and p73, were shown to be involved in autophagic processes [24,25,33-37]. However, to date no evidences were reported that p63 plays a role in autophagic pathway.

ATM is a biosensor that coordinates cellular response to various damaging signals to preserve genomic integrity [8,9,22,23]. ATM has been recently implicated in cellular response to elevated reactive oxygen species (ROS) and therefore involved in redox homeostasis [8,9,22]. The key reports of the Cheryl Walker' research team showed that the ATM import to cytoplasm activates the specific phosphorylation of LKB1 at the Threonine-366 position leading to subsequent TSC2 activation via the LKB1/AMPK metabolic pathway, and reduction of mTOR level, in turn promoting autophagy [8,9].

Our previous observations showed that the cisplatin exposure induced the ATM-dependent phosphorylation of ΔNp63α resulting in the p(S385)-ΔNp63α modification and subsequently leading to a proteasome-dependent degradation of ΔNp63α in SCC cells [4]. Our later studies emphasized the p-ΔNp63α role in transcriptional regulation of numerous gene targets involved in tumor cell response to cisplatin, some of them with pro-apoptotic functions and some - with cell survival functions [5]. The complex response of the p-ΔNp63α dependent gene targets to cisplatin prompted us to continue the quest for the signaling pathways leading to cisplatin sensitivity or cisplatin resistance displayed by tumor cells [3-5]. Recent observations by the research groups of Ted Hupp and Borivoj Vojtesek defined ΔNp63α as a novel regulator of p53 activation through the ATM kinase transcription [7]. They further reported that the ΔNp63α protein interacts with the ATM promoter-derived CCAAT sequence [38], previously shown to be critical for the p-ΔNp63α transcription function in SCC upon cisplatin exposure [5]. Intriguingly, these investigators showed that DNp63α activates the ATM gene transcription, whereas TAp63α does not, highlighting an essential role for the TA2 domain in mediating ΔNp63α function [7].

In this study, we found that p-ΔNp63α binds the ATM promoter, induces the ATM promoter activity and activates the ATM cytoplasmic accumulation. We further found that the p-ΔNp63α protein interacts with the Rpn13 protein leading to a proteasome-dependent degradation of p-ΔNp63α. Next, we observed that ATM triggers the LKB1-AMPK-tuberin pathway leading to a down-regulation of mTOR subsequently enhancing the cisplatin-dependent autophagy in wild type ΔNp63α cells upon cisplatin exposure. Using the SCC cells with an altered ability to support the ATM-dependent ΔNp63α phosphorylation, non-phosphorylated ΔNp63α failed forming protein complexes with Rpn13 and allowing the latter to bind and target LKB1 into a proteasome-dependent degradation pathway thereby modulating a cisplatin-induced autophagy. SCC cells with the innate resistant/impaired response to a cisplatin-induced cell death displayed a greater ratio of non-phosphorylated ΔNp63α/p-ΔNp63α than cells that are sensitive to cisplatin-induced cell death. Based on our findings so far, we suggest that the choice made by Rpn13 between p-ΔNp63α or LKB1 to be targeted for degradation is critical for cell death decision made by cancer cells in response to chemotherapy. The discovery that the ΔNp63 promoter is subject to both p53-mediated activation and repression by ΔNp63α [39], and that ATM-dependent phosphorylation mediates ΔNp63α degradation [4,5] suggests that activity of the damage-response ΔNp63α-ATM-p53 pathway is finely modulated by complex feedback mechanisms [7]. Further dissection of this pathway should provide molecular targets for combating cancer and ageing [7,9,40-44].

## METHODS

### Cells and reagents.

We have used the head and neck squamous carcinoma (SCC) stable cell lines expressing wild type ΔNp63α or ΔNp63α-S385G (with an altered ability to be phosphorylated by ATM kinase) as previously described [4,5]. We also used cisplatin-sensitive (SCC-25) and resistant (SCC-25CP) squamous carcinoma cell lines obtained from Dr. J.S. Lazo (Department of Pharmacology, University of Pittsburgh School of Medicine) as a result of the Material Transfer Agreement [20]. Cells were maintained in RPMI medium 1640, 10% fetal bovine serum. Cells were incubated with 10 μg/ml cisplatin, 25 μM of lactacystin β-lactone (Calbiochem) for indicated periods of time, as described elsewhere [13]. Cells were lysed in 50 mM Tris, pH 7.5, 100 mM NaCl, 2mM EDTA, 0.5% Triton X-100, 0.5% Brij-50, 1 mM PMSF, 0.5 mM NaF, 0.1 mM Na_3_VO_4_, 2X protease inhibitor cocktail, sonicated for 10 sec time intervals, and clarified for 30min at 15,000xg. Supernatants (total lysates) were used for immunoprecipitation and immunoblotting [4,5,13]. Control (scrambled) siRNA and Rpn13 siRNA (sc-72453) were obtained from Santa Cruz Biotechnology, while siRNA against LKB1 was purchased from Dharmacon [45]. SiRNAs (200 pmol/six-well plate) were transiently transfected into cells using FuGENE 6 (4 μL, Roche) for 24h and then after the 24h treatment with control media or 10μg/ml cisplatin.

### Isolation of cytoplasmic fraction.

1-2 × 10^6^ cells were resuspended in a hypotonic lysis buffer (10 mM HEPES pH 7.9, 10 mM KCl, 0.1 mM EDTA, 0.1 mM EGTA) with protease inhibitors (Sigma), Triton X-100 (0.6% final concentration) was then added and the nuclei were pelleted at 2,500-3,000xg for 10 min at 4°C. Supernatants served as cytoplasmic fractions [13].

### Antibodies.

We used a rabbit polyclonal antibody against ΔNp63α (Ab-1, EMD Chemicals), a mouse monoclonal antibody against β-actin (Sigma), and rabbit polyclonal antibodies against UCH3 (ab38528), mTOR (ab2833) and Rpn13 (ab91567), and a mouse monoclonal antibody against ATM (2C11A1, ab78) were purchased from Abcam. We also obtained a mouse monoclonal antibody against Rpn13 (M01, clone 3C6, Abnova). A mouse monoclonal anti-LKB1 antibody (clone 27D10, ab3050) was obtained from Cell Signaling Technology. A custom rabbit polyclonal antibody against p ΔNp63α (ATM motif, residues 379-392) was previously described [4]. A polyclonal rabbit anti-phospho-tuberin antibody (TSC2-S1387, AP3338a; which represents the AMPK-dependent phosphorylation) was obtained from Abgent.

### Chromatin immunoprecipitation (ChIP).

First, 5×10^6^ cell equivalents of chromatin (2-2.5 kb in size) were immunoprecipitated (IP) with 10 μg of anti-p ΔNp63α antibody as described elsewhere [5,13]. After reversal of formaldehyde crosslinking, RNAase A and proteinase K treatments, IP-enriched DNAs were used for PCR amplification. PCR consisted of 40 cycles of 94°C for 30 s, 60°C for 30 s, and 72°C for 30 s using Taq DNA polymerase (Invitrogen). The following PCR primers were used for amplification of the ATM promoter: for the specific region, sense, (−920) 5'- TTCAGGGGTCCTAATTAAGT −3'(901), and antisense, (−570) 5'- TGATCAAAACCACAGCAGG-3' (−551) yielding the 350 bp PCR product, and for the non-specific region, sense, (−2000) 5'- TAGGGGTGATTCTGCCCTCC-3' (−1880) and antisense, (−1821) 5'- AATTATGAGGCCCAAAATG-3' (−1802) yielding the 199 bp PCR product. Binding of the endogenous p-ΔNp63α protein to the ATM promoter was also assessed by qPCR using the above-mentioned primers for the specific region of the ATM promoter as previously described [13]. ChIP-PCR values (relative units, RU) were normalized by the GAPDH values and those obtained from the control samples (cells treated with control medium) were designated as 1. Experiments were performed in triplicate.

### Luciferase reporter assay.

We used the pGL3-ATM (S118526, SwitchGear Genomics) promoter-luciferase reporter plasmid (encompassing −1259 bp to +1 bp of the ATM promoter). A total of 5x10^4^ cells/well in a 24-well plate were transfected with 100 ng of the pGL3 luciferase reporter constructs plus 1 ng of the Renilla luciferase plasmid pRL-SV40 (Promega) using FuGENE 6 (Roche) as previously described [3,13]. At 24h, cells were also treated with 10 μg/ml cisplatin or control medium and then after an additional 24h, luciferase assays were performed using the Dual luciferase reporter assay kit (Promega). For each experiment, the wells were transfected in triplicate and each well was assayed in triplicate by measuring the Firefly luciferase activity in a luminometer. Renilla luciferase activity was measured in the same tube [3,13]. The values for Firefly luciferase activity were normalized against the Renilla luciferase activity values for each transfected well. Resulting data were presented as relative luciferase units (RLU).

### Autophagy assay.

Cells were transiently transfected with scrambled siRNA, siRNA against Rpn13 or LKB1. Cells were exposed to control medium or 10 μg/ml cisplatin for 24h in the presence of lyzosomal protease inhibitors (10μg/ml of both E64d and pepstatin A purchased from Sigma) as previously described [19]. Protein levels of LC3B-I and LC3B-II were tested with a rabbit polyclonal antibody against MAP LC3α/β (LC3B, L7453, Sigma Aldrich Co). Immunoblots were scanned using PhosphorImager (Molecular Dynamics) and quantified by ImageQuant software version 3.3 (Molecular Dynamics). Values of LC3B-II were expressed as a portion of LC3B-I values defined as 1. The LC3B-II/LC3B-I ratios were plotted as bars using the Microsoft Excel software with standard deviations (± SD) resulting from three independent experiments and three individual measurements of each experiment.

### Immunofluorescence microscopy.

Cells were washed with ice-cold phosphate-buffered saline and after fixation with 4% paraformaldehyde for 10 min at room temperature, they were permeabilized with 50μg/ml digitonin for 5 min. Cells were then quenched in 0.1% sodium borohydride for 5 min, and blocked with 10% goat serum, 1% bovine serum albumin (BSA, Amersham Biosciences) at room temperature for 60 min. Cells were incubated overnight with the primary antibody against MAPLC3α/β diluted in 1% BSA at 4°C. After washing, the cells were incubated with the Cy3-conjugated anti-rabbit antibody (1:500, Jackson ImmunoResearch Laboratories Inc) diluted in 1% BSA for 1h. Finally, images were obtained using a Leica TCS-NT laser scanning microscope system and processed with Adobe Photoshop software [29-31].

### Statistical analysis.

The data represent mean ± SD from three independent experiments and the statistical analysis was performed by Student's t test at a significance level of p<0.05 to <0.001.

## SUPPLEMENTAL FIGURES

Supplemental Figure S1. Quantitative PCR analysis of the ChIP binding. Wild type ΔNp63α cells and ΔNp63α-S385G cells were treated with the control medium (CIS, −) or 10μg/ml cisplatin (CIS, +) for 24h. ChIP assay of ATM promoter was performed with antibodies against p-ΔNp63α (black) and ΔNp63α (grey). The quantitation of binding was monitored by qPCR using the following specific ATM promoter primers: sense, (−920) 5'- TTCAGGGGTCCTA-ATTAAGT −3'(901), and antisense, (−570) 5'- TGATCAAAACCACAGCAGG-3' (−551) yielding the 350 bp PCR product. ChIP-PCR values (relative units, RU) were normalized by the GAPDH values and those obtained from the control conditions (cells treated with control medium) were designated as 1. Experiments were performed in triplicate. Numerical values indicate the fold differences between control conditions and cisplatin treatment conditions.

Supplemental Figure S2. Expression levels for ΔNp63α, p-ΔNp63α and LKB1 in SCC-25 cells and SCC-25CP cells upon cisplatin exposure. Cells were treated with the control medium (CIS, −) or 10μg/ml cisplatin (CIS, +) for 24h. Immunoblotting of total lysates was performed with indicated antibodies and loading level was monitored by the β-actin level.
