# Supplementary figures and images for: Phospho-ΔNp63α/Rpn13-dependent regulation of LKB1 degradation modulates autophagy in cancer cells
Source: Aging (Albany NY). 2010 Dec 20;2(12):959–68. doi: 10.18632/aging.100249 (PMC3034184; doi:10.18632/aging.100249)

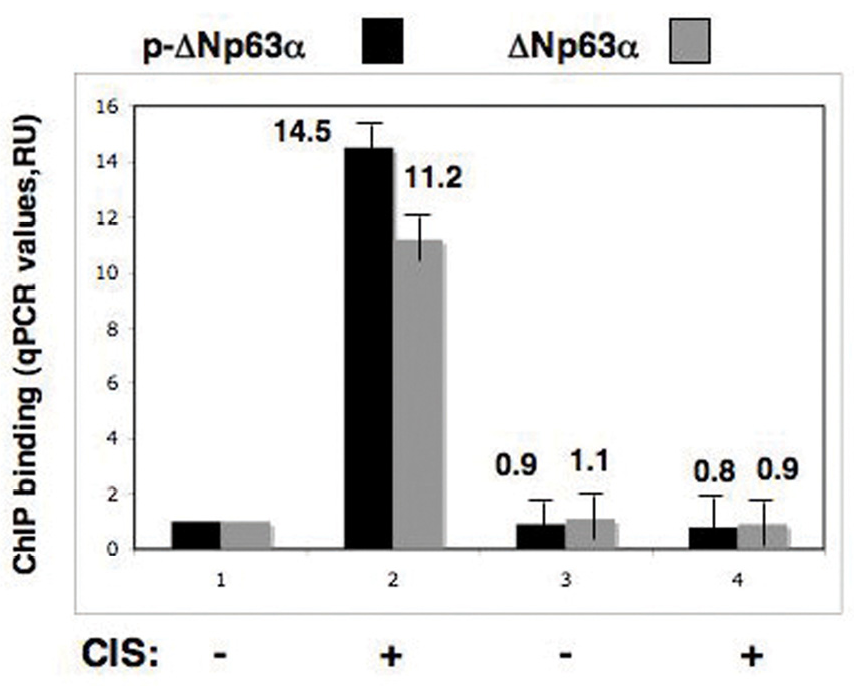

Supplement: Supplemental Figure S1. — Wild type ΔNp63α cells and ΔNp63α-S385G cells were treated with the control medium (CIS, −) or 10μg/ml cisplatin (CIS, +) for 24h. ChIP assay of ATM promoter was performed with antibodies against p-ΔNp63α (black) and ΔNp63α (grey). The quantitation of binding was monitored by qPCR using the following specific ATM promoter primers: sense, (−920) 5'- TTCAGGGGTCCTA-ATTAAGT −3'(901), and antisense, (−570) 5'- TGATCAAAACCACAGCAGG-3' (−551) yielding the 350 bp PCR product. ChIP-PCR values (relative units, RU) were normalized by the GAPDH values and those obtained from the control conditions (cells treated with control medium) were designated as 1. Experiments were performed in triplicate. Numerical values indicate the fold differences between control conditions and cisplatin treatment conditions. [file aging-02-959-s001.tif]

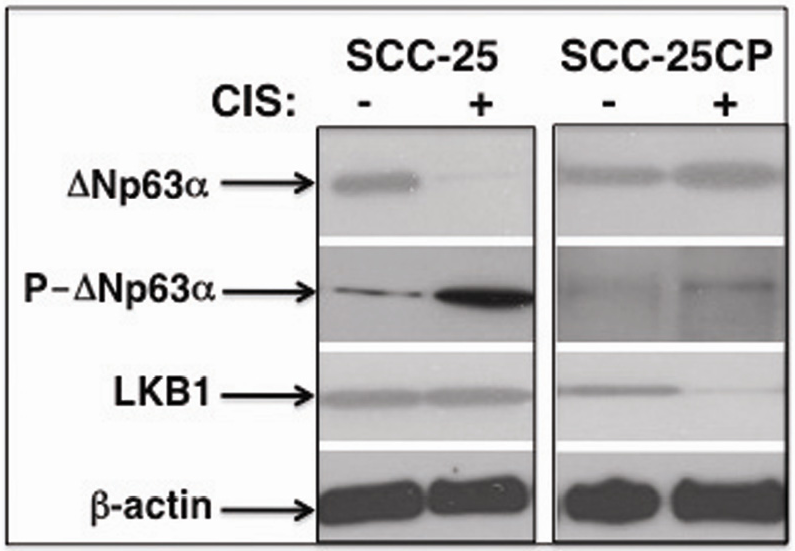

Supplement: Supplemental Figure S2. — Cells were treated with the control medium (CIS, −) or 10μg/ml cisplatin (CIS, +) for 24h. Immunoblotting of total lysates was performed with indicated antibodies and loading level was monitored by the β-actin level. [file aging-02-959-s002.tif]
